# Adsorption and bonding strength of chromium species by ferrihydrite from acidic aqueous solutions

**DOI:** 10.7717/peerj.9324

**Published:** 2020-06-11

**Authors:** Agnieszka Dzieniszewska, Joanna Kyziol-Komosinska, Magdalena Pająk

**Affiliations:** Institute of Environmental Engineering, Polish Academy of Sciences, Zabrze, Poland

**Keywords:** Ferrihydrite, Chromium species, Adsorption, Isotherms, Mobility

## Abstract

The adsorption behavior of Cr(III) and Cr(VI) ions onto laboratory-synthesized 2-line ferrihydrite was investigated under a batch method as a function of initial chromium concentration (0.1–1000 mg L^−1^) and pH (3.0 and 5.0). Moreover, the effect of the type of anion (chloride and sulfate) on Cr(III) adsorption was studied. The affinity of Cr(III) ions for the ferrihydrite surface depended on both the type of anion and pH of the solution and the maximum adsorption capacities decreased as follows: *q* (SO_4_^2−^, pH 5.0) > *q* (SO_4_^2−^, pH 3.0) > *q* (Cl^−^, pH 5.0) > *q* (Cl^−^, pH 3.0), and were found to be 86.06 mg g^−1^, 83.59 mg g^−1^, 61.51 mg g^−1^ and 40.67 mg g^−1^, respectively. Cr(VI) ions were bound to ferrihydrite in higher amounts then Cr(III) ions and the maximum adsorption capacity increased as the pH of the solution decreased and was 53.14 mg g^−1^ at pH 5.0 and 83.73 mg g^−1^ at pH 3.0. The adsorption process of Cr species was pH dependent, and the ions were bound to the surface of ferrihydrite by surface complexation. The Sips isotherm was the best-fit model to the results obtained from among the four isotherm models used, i.e., Freundlich, Langmuir, Dubinin-Radushkevich and Sips, indicating different adsorption centers participate in Cr uptake. In order to assess the bonding strength of the adsorbed chromium ions the modified BCR procedure, dedicated to the samples with a high iron content, was used. The results of the sequential extraction showed that Cr(III) ions were bound mainly in the immobile residual fraction and Cr(VI) ions were bound in the reducible fraction. The presence of Fe (oxyhydr)oxides in soil and sediments increases their adsorption capacity for Cr, in particular for hexavalent Cr in an acid environment due to their properties (high pH_PZC_).

## Introduction

The quality of the environment is constantly deteriorating due to the increasing number of applications of heavy metals as modern science and technology develops. The industrial discharge consisting of major toxic heavy metal and metalloid ions poses a serious hazard to the environment and affects water quality. Heavy metals are not biodegradable and some of them (lead, cadmium, mercury) are toxic even at trace levels, while other heavy metals are essential nutrients (cobalt, zinc, iron) but can be toxic in larger amounts or in certain forms. Hence, their concentration in industrial wastewater should be reduce below acceptable levels before discharging (Bedemo, Chandravanshi & Zewge, 2016).

One of the widely and commonly used metals is chromium (Cr). Cr is used in many industrial processes such as electroplating, steel fabrication, leather tanning, petroleum refining, water cooling, mining, pulp and paper production, dyes and pigments, film and photography and wood preservation (Bellú et al., 2008; Bhattacharya et al., 2008; Saha & Orvig, 2010), from which it is released to various environmental elements, including air, soil and aquatic environment.

The toxicity and behavior of Cr in the soil-water environment depends on its speciation, pH-Eh system and the presence of ligands (Ryan, Hillier & Wall, 2008; Ajouyed et al., 2010; Kyziol-Komosinska et al., 2018).

Cr can exist in several oxidation states; however, in the environment it occurs mainly in most stable trivalent cationic Cr(III) and hexavalent anionic Cr(VI) forms, which differ in the physicochemical properties and in the chemical and biochemical reactivity (Kotaś & Stasicka, 2000). In the trivalent state Cr is a macronutrient essential for health and is known to regulate carbohydrates, lipids, and proteins, but excess Cr(III) can cause adverse health effects such as skin rashes (Valle et al., 2017). In contrast, hexavalent Cr is highly toxic, carcinogenic and mutagenic to the living organisms due to its high water solubility, mobility and easy reduction (Asgari et al., 2008; Saha & Orvig, 2010). Scientific studies have shown that Cr(VI) causes skin irritation, ulcer formation, kidney and liver damage and respiratory organs diseases (Anwar et al., 2009; Gupta & Babu, 2009). However, Cr(III) can be oxidized to the toxic hexavalent form, e.g., in the presence of manganese (Saha & Orvig, 2010) or under oxidative conditions. In natural waters, the change in Cr(III) speciation can be affected by the presence of complex-forming ligands or extensively Cr hydrolysis at pH values characteristic of soil solutions (Csoban & Joo, 1999). Ligands can change the ability of ions to interact with the surface of solids and affect the type of possible bonds by influence the electric charge and radius of an ion (i.e., the Cartledge ionic potential) (Kyziol-Komosinska et al., 2014).

Cr is regarded (i.e., by the Environmental Protection Agency, USA) as priority-toxic pollutant. According to The European Drinking Water Directive (Council Directive, 1998), the content of Cr in drinking water should not exceed 0.05 mg L^−1^. According to current Polish legislative regulations of the Ministry of the Environment (Regulation of the Minister of the Environment, 2016), the maximum allowed Cr concentration in surface water should not exceed 0.05 mgL^−1^ for total Cr and 0.02 mg L^−1^ for Cr(VI). Moreover, the water quality standards for sewage introduced into water or to the ground (Ministry of the Environment of Poland, 2014) state, that concentrations of Cr(VI) ions and total Cr, although depending on the kind of industry, should be lower than 0.05–0.5 mg L^−1^ and 0.5–1.0 mg L^−1^, respectively.

The negative influence of Cr on human health and exceeded concentrations of Cr in the soil/water system and wastewater, has become a driving force for research on its behavior in soil (Choppala, Bolan & Park, 2013), as well as effective methods of water and wastewater treatment.

The binding and mobility of Cr ions in the environment depend on the type of minerals and organic matter that create the soil, river suspension and sediments (Kyziol, Twardowska & Schmitt–Kopplin, 2006; Merdy, Gharbi & Lucas, 2009; Kyziol-Komosinska et al., 2010; Dos Anjos et al., 2014). The most effective minerals in metal uptake are secondary minerals, such as clay minerals (kaolinite, montmorillonite, illite) and iron (oxyhydr)oxides (ferrihydrite, goethite), forming during the weathering of primary minerals (Ugwu & Igbokwe, 2019). The ubiquity and abundance of iron oxides in the subsurface highlight their important roles in influencing the fate and transport of heavy metals (Wang et al., 2019). Iron (oxyhydr)oxides exist as a surface coating mineral particles modifying their physico-chemical properties or individual particles.

One of the most reactive and most common iron oxides, which occurrence has been reported at near-neutral pH conditions in soils, sediments, and water bodies, is ferrihydrite. In addition to its natural presence in the environment, ferrihydrite can also occurs as a result of human activities such as acid mine drainage (Antelo, Arce & Fiol, 2015).

Ferrihydrite (Fh) has significant retention effects on the migration of contaminants in both anionic and cationic forms due to its properties like small particle size, large specific surface area and high value of the point of zero charge (pH_*PZC*_∼8.0) (Meng et al., 2012; Kumar et al., 2014; Hu et al., 2019). In an acidic and neutral medium, at pH < 8.0, its surface is positively charged and in a basic medium, at pH > 8.0, the surface has a negative charge (Ajouyed et al., 2010).

Ferrihydrite exists as nano-crystals. The low degree of order causes difficulties in determining its structure, therefore several different structures of ferrihydrite have been proposed (Cornell & Schwertmann, 2003). Research conducted by Michel et al. (2007) showed that three types of Fe sites existst in the Fh structure, i.e., Fe1 and Fe2 in hexa-coordination and Fe3 in tetrahedral coordination. Hiemstra (2013) suggested that horizontal sheets with octahedra Fe1 are sandwiched by octahedral Fe2 and tetrahedral Fe3. According to Hiemstra (2013) studies Fe2 octahedra and Fe3 tetrahedra are not contributing to the formation of the surface groups. Only the layers with Fe1 octahedra have singly-coordinated surface groups that form two types of ≡FeOH binding sites. The Fe1 octahedra layers form alternating rows, the first of which allows the formation of double-corner complexes (e.g., with anions such as SeO${}_{3}^{2-}$ or AsO${}_{4}^{3-}$), and the second one enables the formation of bidentate edge surface complexes (found for instance for UO${}_{2}^{2+}$). The metal ion binding functional groups can be protonated/deprotonated according to the following Reactions Eqs. (1) and (2) (Dzombak & Morel, 1990):

(1) \begin{eqnarray*}\equiv \mathrm{FhOH}+{\mathrm{H}}^{+}\rightarrow \equiv {\mathrm{FhOH}}_{2}^{+} \log \nolimits K=7.29\end{eqnarray*}

(2) \begin{eqnarray*}\equiv \mathrm{FhOH}\rightarrow \equiv {\mathrm{FhO}}^{-}+{\mathrm{H}}^{+} \log \nolimits K=-8.93\end{eqnarray*}

where ≡Fh indicates a ferrihydrite surface species.

As ferrihydrite is an effective barrier to the migration of heavy metal ions in soils and aqueous systems, it can be a potential adsorbent for pollutants uptake in the pH range of most natural waters (Ajouyed et al., 2010). Among the available adsorbents, natural and synthetic iron oxides and (oxyhydr)oxides (Asgari et al., 2008; Campos, 2009; Ajouyed et al., 2010; Adegoke et al., 2014; Shahriari et al., 2014; Kar & Equeenuddin, 2019), including ferrihydrite (Zachara et al., 1987; Tzou, Wang & Loeppert, 2003; Zhu et al., 2010; Pieczara & Rzepa, 2016), were examined for Cr removal.

Among different interactions and processes with soil, including adsorption–desorption, dissolution-precipitation, and transformation, adsorption is a basic and fundamental process, which controls the mobility, bioavailability, and toxicity of heavy metals. Moreover, adsorption is one of the most effective methods of Cr removal from water and wastewater (Sarin & Pant, 2006; Bellú et al., 2008; Bhattacharya et al., 2008; Anwar et al., 2009). The significant advantages of this technique are high efficiency, selectivity, simple technological requirements, low operating cost as the low cost adsorbents are used, minimization of chemical or biological sludge, some of the adsorbent can be regenerated or simplify confined in controlled disposal systems (Hajji et al., 2019).

The aim of this study was to determine the adsorption behavior of Cr(III) and Cr(VI) ions onto synthetic ferrihydrite. The effect of initial concentration (0.1–1,000 mg L^−1^), pH (3.0 and 5.0) and the type of anion for Cr(III) ions (chloride and sulfate) on the adsorption capacity were investigated. It is very important to study the adsorption capacity under acidic conditions because some sewage and leachate from landfills have pH < 6. Four adsorption isotherms (Freundlich, Langmuir, Dubinin-Radushkevich and Sips) were used to understand the nature of the adsorption process and the estimated parameters allowed determination of the maximum adsorption capacity and the mechanism of ions binding. Additionally, the bonding strength of adsorbed ions and thus their mobility was determined, which allows prediction of the behavior of metal ions in the soil-water environment and or the procedure for handling spent adsorbents.

## Materials and Methods

### Preparation and characterization of ferrihydrite

Ferrihydrite (Fh) was synthesized by precipitation of iron(III) nitrate nonahydrate (Fe(NO_3_)_3_⋅9H_2_O) in an alkaline medium (1 M KOH), according to the procedure described by Schwertmann & Cornell (2000) and Cornell & Schwertmann (2003).

The ferrihydrite sample was characterized by scanning electron microscopy coupled with energy-dispersive X-ray spectroscopy (SEM-EDS), X–ray powder diffraction (XRD), Raman spectroscopy and low-temperature nitrogen adsorption.

Observations of the ferrihydrite micromorphology and chemical analyses in the micro area were carried out using a FEI Quanta 200 FEG scanning electron microscope (SEM) with EDS/EDAX attachment. The accelerating voltage was set to 20 kV and the low-vacuum mode was applied. The sample was analyzed without coating.

X-ray powder diffraction analysis was carried out with a Philips APD PW 3020 X’Pert diffractometer equipped with graphite monochromator using Cu-Kα radiation. The XRD patterns were recorded in the range of 2–73^∘^2θ, with a 0.05^∘^2θ step.

The Raman spectra were collected using a Thermo Scientific DXR Raman Microscope equipped with a 780 nm wavelength laser. The spectra were recorded in the range of 100–3,000 cm^−1^. The conditions of the preparation and analysis have been selected to prevent the transformation of ferrihydrite to hematite during spectrum recording (Mazzetti & Thistlethwaite, 2002; Hanesch, 2009).

The specific surface area, total pore volume and average pore diameter were determined by N_2_ adsorption and desorption isotherms at liquid nitrogen temperature (77 K) using ASAP 2020 (Micromeritics) surface area analyzer. The sample was degassed under vacuum at 90 °C for 24 h prior to analysis. These conditions were selected to avoid the thermal transformation of ferrihydrite (Weidler, 1997; Glasauer et al., 2000; Pieczara, Rzepa & Zych, 2013).

The point of zero charge (pH_*PZC*_) of ferrihydrite was determined according to the method described by Calvete et al. (2009). The pH in mineral-water suspension (1:10 ratio) was carried out using a pH meter with a combination pH electrode (glass membrane electrode and a reference electrode) (ERH-111 Hydromet, Poland). The pH meter (Elmetron, Poland) was calibrated with standard buffer solutions (Merck, Germany).

Cation exchange capacity (CEC) of ferrihydrite was assessed with the method described by Thomas (1982). This method consists in extracting the exchangeable acidity (H^+^ and ionic Al species) with 1 M KCl solution and subsequent titration with 0.1 M NaOH solution to convert the entire Al to Al(OH)_3_ (Bélanger et al., 2006).

### Preparation of chromium solutions

The stock solutions of 1,000 mg L^−1^ of Cr(III)-Cl, Cr(III)-SO_4_ and Cr(VI) were prepared by dissolving appropriate weighed quantity of chromium chloride (CrCl_3_.6H_2_O), chromium sulfate (KCr(SO_4_)_2_ ⋅12H_2_O) and potassium chromate (K_2_CrO_4_) in 1,000 mL of water. The pH values of the stock solutions were: 2.85, 3.02 and 8.52, respectively.

The working solutions at the desired concentrations (0.1–1,000 mg L^−1^) were prepared from the stock solutions by making successive dilutions. The pH of the Cr solutions was adjusted to 3.0 and 5.0 by adding 0.1 M KOH solution to Cr(III) solutions or 0.1 M H_2_SO_4_ solution to Cr(VI) solution, to reflect acidic solutions.

Reduction potential (Eh) was measured in an initial Cr(VI) solution at *C*_0_ of 1,000 mg L^−1^ at pH 3.0 and 5.0 immediately after preparation and after 24 h and directly in the equilibrium solutions after adsorption using a Redox combination electrode consisting of a platinum sensor and an Ag/AgCl reference electrode (ERPt-13 Hydromet, Poland).

All the chemicals used were analytical grade. Ultrapure water of resistivity of 18.2 MΩcm (obtained from MilliQ system—Millipore, Billerica, USA) was used in all experiments.

### Adsorption experiments

The adsorption process of Cr species onto ferrihydrite was investigated using a batch method in the wide range of initial Cr concentrations (0.1–∼1,000 mg L^−1^) at pH 3.0 and 5.0 and adsorbent dose of 1 g L^−1^ (i.e., the solid phase to solution ratio was 1:1000) at room temperature (23 ± 2 °C). Study of Cr(III) uptake from chloride and sulfate solutions allowed determination of the influence of the type of anion on the adsorption capacity. The prepared suspensions were agitated on a horizontal shaker (the intensity of agitation was 2 rps) for 24 h to reach equilibrium. Afterwards, the solutions were centrifuged (Avanti J25, Beckman Coulter) at 10,000 rpm for 20 min to separate the solution from the solid phase. To determine initial and equilibrium concentrations of Cr(III) and Cr(VI) in the solutions atomic absorption spectrophotometry (Avanta, GBC) was used. Moreover, the pH in the equilibrium solutions was measured.

In each series of measurements, two kinds of quality control experiments were carried out: (1) blank experiment with the adsorbent and working solutions, but without addition of Cr, and (2) two experiments with various concentrations of Cr in working solutions, but without the adsorbent.

The adsorption capacity and the removal efficacy of Cr(III) and Cr(VI) from the aqueous solutions were calculated using the following Eqs. (3) and (4), respectively:

(3) \begin{eqnarray*}q= \frac{ \left( {C}_{0}-{C}_{eq} \right) \cdot V}{m\cdot 1000} ({\mathrm{mg~ g}}^{-1})\end{eqnarray*}

(4) \begin{eqnarray*}RE= \frac{ \left( {C}_{0}-{C}_{eq} \right) }{{C}_{0}} \cdot 100(\text{%})\end{eqnarray*}

where *q* is the amount of Cr(III) or Cr(VI) adsorbed per unit mass of ferrihydrite (mg g^−1^), *RE* is the removal efficacy (%), *C*_0_ and *C*_*eq*_ are the initial and equilibrium concentrations of Cr(III) or Cr(VI), respectively (mg L^−1^), *V* is the volume of the solution (mL), *m* is the mass of the adsorbent (g).

All experiments were performed in triplicate. Microsoft Excel 2010 software was used to calculate means and standard deviations (SD).

### Adsorption isotherm models

One of the possibilities to describe the adsorption behavior under different experimental conditions is to use mathematical models, which are very useful e.g., in process optimization. Many models of varying degrees of complexity have been developed to describe adsorption systems. In this study the equilibrium data obtained from the batch experiments were analyzed by one of the most frequently used adsorption models, i.e.

–Freundlich—the empirical model that describes mutlilayer adsorption on heterogeneous surfaces Freundlich (1906), where the parameter 1/n_*F*_ is a measure of the surface heterogeneity, which becomes more heterogeneous as its value gets closer to zero. The value of 1/n_*F*_ < 1 implies a chemisorption process and 1/n_*F*_ > 1 indicates cooperative adsorption (Foo & Hameed, 2010);

–Langmuir –assumes monolayer adsorption on homogeneous surfaces with energetically equivalent adsorption sites, without interactions between the adsorbate molecules and it allows estimation of the maximum adsorption capacity of the adsorbent Langmuir (1916);

–Dubinin-Radushkevich –an empirical model that describes adsorption with a Gaussian energy distribution onto heterogeneous surfaces Dubinin (1960);

–Sips –a three-parameter empirical model is a combination of the Langmuir and Freundlich isotherms describing adsorption on heterogeneous surfaces. At low adsorbate concentrations it is reduced to the Freundlich isotherm and at high concentrations the equation is transformed into the Langmuir isotherm, predicting monolayer adsorption Sips (1948).

The equations are summarized in Table S1.

Based on the value of the parameter K_*L*_ estimated from the Langmuir equation (Eq. S1.1), it is possible to calculate the separation factor R_*L*_, from Eq. (5):

(5) \begin{eqnarray*}{R}_{L}= \frac{1}{1+{K}_{L}{C}_{0}} \end{eqnarray*}

The value of the parameter R_*L*_ indicates the shape of isotherm. The process is unfavorable if R_*L*_ > 1, linear if R_*L*_ = 1, favorable if 0 < R_*L*_ < 1 and irreversible if R_*L*_ = 0 (Foo & Hameed, 2010).

The parameter *β* (Eq. S3.1), on the other hand, allows calculation of the mean free energy (*E)* of adsorption per molecule of the adsorbate:

(6) \begin{eqnarray*}E= \frac{1}{\sqrt{2\beta }} \end{eqnarray*}

The value of *E* in the range of 8–16 kJ mol^−1^ indicates the adsorption is a chemical process, whereas if the value is lower than 8 kJ mol^−1^, the adsorption process is of a physical nature (Das et al., 2019).

The parameters estimated from selected models provide some insights into the mechanism of the adsorption process, the surface properties and the affinity of the adsorbent (Vaghetti et al., 2009). The nonlinear regression method using the Lavenberg-Marquardt algorithm (Statistica ver. 9.0) as well as linear regression method were applied to determine the values of the parameters in the adsorption isotherms.

Three error functions, sum of the squares of the errors *SSE*, residual root mean square error *RMSE* and nonlinear chi-square test *χ*^2^ (Table S2), as well as the coefficient of determination *R*^2^ were applied to evaluate the goodness of fit of the isotherm models to the experimental results. Error functions measure the differences between the experimental and the calculated from mathematical models data, and their low values indicate a good fit of the model to the obtained results (Foo & Hameed, 2010, Terdputtakun et al. (2017)).

### Mobility and bioavailability of adsorbed chromium ions

Bonding strength, and thus the mobility and bioavailability of Cr ions adsorbed onto ferrihydrite were evaluated by a modified three-step The European Community Bureau of Reference (BCR; now the Standards, Measurements and Testing Programme) sequential extraction procedure, which was designed to examine the potential for metal release under certain environmental conditions, such as ion exchange, reduction and oxidation (Ryan, Hillier & Wall, 2008).

The studies on the mobility of metals bound to iron and manganese oxides indicate that the modified method should be used in this study (Kaasalainen & Yli-Halla, 2003; Joksic et al., 2005; Tokalioglu & Kartal, 2005). The modified procedure differs from the original one in the higher concentration and lower pH of the reagent in E2 (Table S3). It was observed that modified reagent provides better attack on the iron (oxyhydr)oxide phase, whereas the original reagent attacked only hydrous oxides of manganese (Mossop & Davidson, 2003).

The procedure was preceded by a water desorption test with ultrapure water at the water to solid phase ratio of 10:1 (E0) (in accordance with the methodology for leaching soluble components from granular wastes and sludges) (EN 12457/1-4, 2002), and after completion the sequential extraction procedure, the residual Cr content (E4) was determined.

For monomineral materials, the metal distribution in individual fractions provides information on the main binding sites and the binding strength to the mineral and helps to determine the susceptibility to the release of the adsorbed metal—from weakly bond (E0–E1) to very strong bond (E4).

The modified BCR sequential extraction was performed in triplicate for the ferrihydrite samples with adsorbed Cr(III) and Cr(VI) ions from the solutions at pH 3.0 and 5.0 with initial concentrations of 10 and 500 mg L^−1^. The Cr concentration after each step were analyzed with an atomic absorption spectrophotometer (Avanta, GBC).

Statistical analysis of the obtained results was performed using Statistica v. 9.0 software. The one-way analysis of variance (ANOVA) together with Tukey’s HSD test were applied to determine the significant differences (*P* < 0.05) among different treatments.

## Results

### Characterization of synthetic ferrihydrite

**Figure 1:**
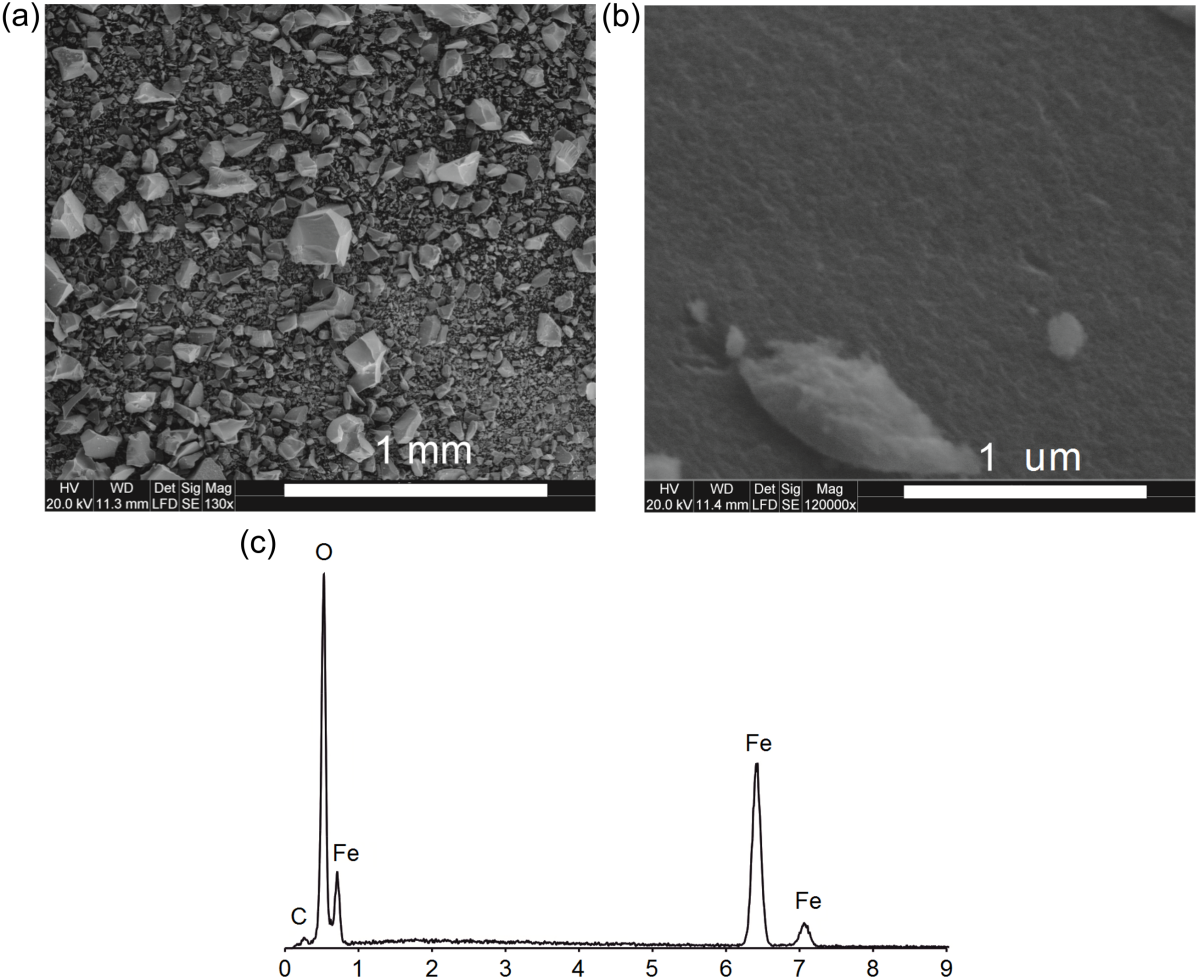
The SEM images: the overall picture of the sediment (A), the surface of the ferrihydrite aggregate (B) and EDS spectrum of ferrihydrite (C).

SEM images show homogeneous material without other surface precipitates (Figs. 1A, 1B). It shows a strong degree of aggregation, typical for ferrihydrite (Fig. 1A).

**Figure 2:**
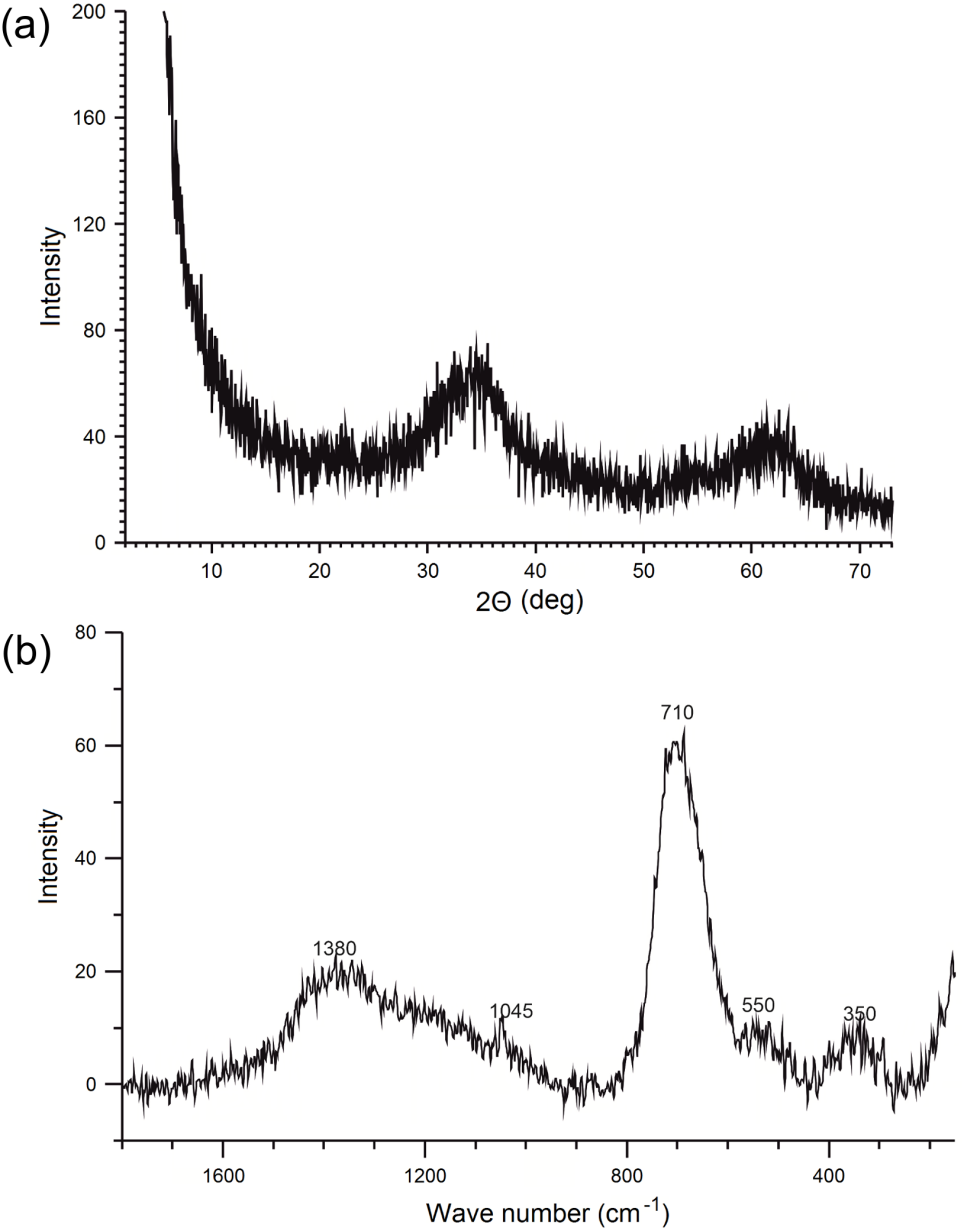
X-ray diffractogram (A) and Raman spectrum (B) of ferrihydrite.

The XRD diffractogram of synthesized ferrihydrite is presented in Fig. 2A and it shows two broad peaks at about 2θ = 34.4^∘^ and 62.8^∘^, which were assigned to the (110) and (115) planes of 2-line ferrihydrite (interplanar distances, d_hkl_ was 0.25 Å  and 0.15 nm) (Cornell & Schwertmann, 2003). At the same time, no characteristic peaks from impurities were detected.

The Raman spectrum of the ferrihydrite sample is presented in Fig. 2B. The Raman spectrum show a strong band with a maximum of about 700 cm^−1^ and weaker bands at about 350, 550 and 1,400 cm^−1^. These are typical features of ferrihydrite (Mazzetti & Thistlethwaite, 2002; Hanesch, 2009; Das & Hendry, 2011). Sharper, but not very strong peak at 1,045 cm^−1^ (Fig. 2B) is the result of the presence of nitrates and is often observed in the spectra of ferrihydrites synthesized by this method (Müller, Ciminelli & Dantas, 2010; Das & Hendry, 2011).

The characteristics of the porous texture of ferrihydrite, as well as the value of the pH, the point of zero charge and CEC are shown in Table 1.

The specific surface area (296 m^2^ g^−1^) of the tested material corresponds very well with the results usually obtained for ferrihydrite (Schwertmann & Cornell, 2000; Cornell & Schwertmann, 2003; *Schwertmann, Friedl & Kyek, 2004; Pieczara, Rzepa & Zych, 2013)*. The pore diameter of Fh was 1.89 nm and indicated that material was dominated by micropores. The total pore volume was 0.175 cm^3^ g^−1^ (Kyziol-Komosinska et al., 2020).

The point of zero charge (pH_*PZC*_) was 7.35 and indicated that at a solution pH lower than 7.35 the surface of ferrihydrite was positively charged and at a solution pH higher than the pH_*PZC*_ value the surface has negative charge.

The value of pH of ferrihydrite-water suspension was 3.90.

CEC of ferrihydrite was very low (0.0930 mmol_+_ g^−1^) (Table 1). Due to the lack of the aluminum compounds in the studied mineral, the entire exchangeable acidity value was constituted by the H^+^ concentration.

### Adsorption of Cr(III) ions on ferrihydrite

**Figure 3:**
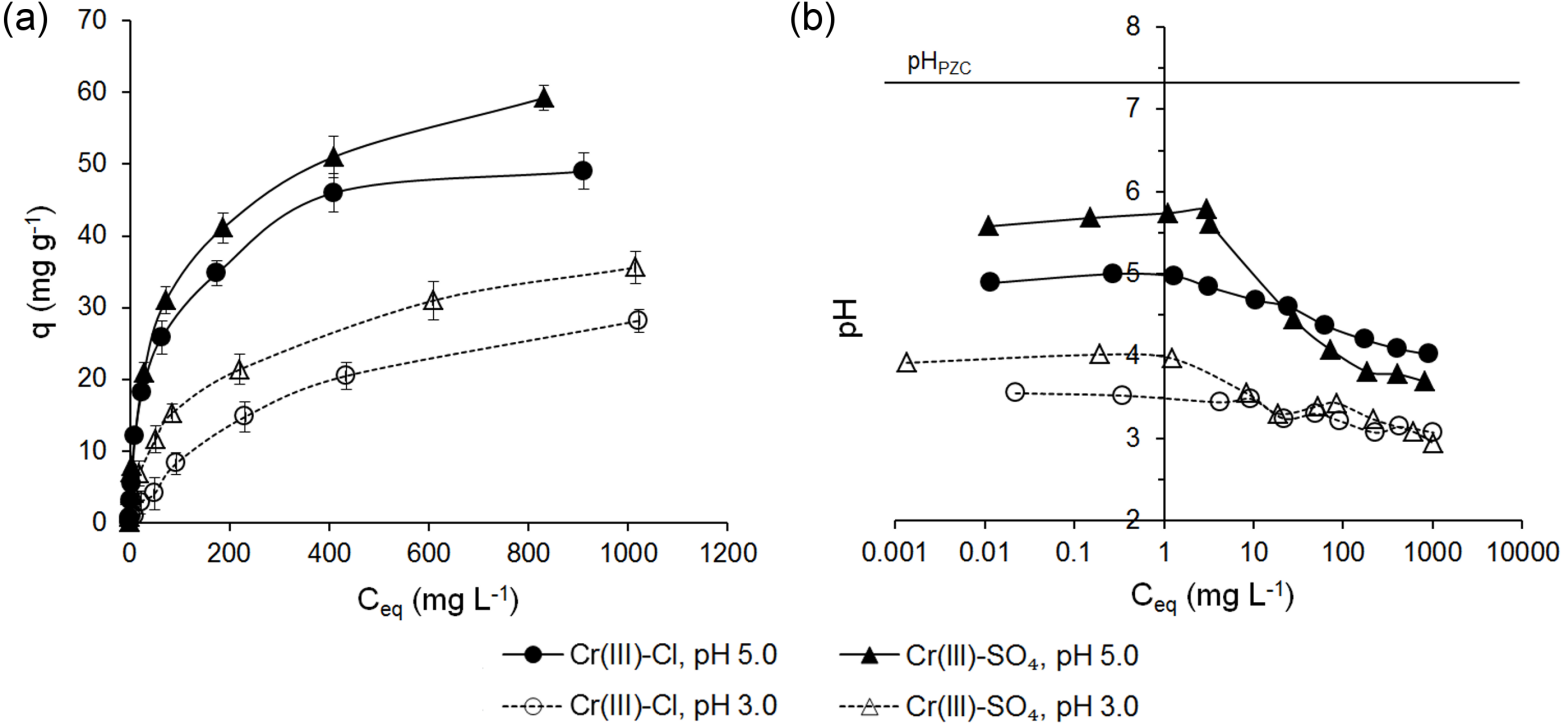
Adsorption of Cr(III) ions on ferrihydrite (A) and the pH values in the equilibrium solutions (B).

The adsorption of Cr(III) ions on ferrihydrite have been studied taking into account an initial concentration of Cr(III) ions, the type of anion and the pH of the solution. The adsorption capacity (*q*) as a function of equilibrium concentration (C_*eq*_) and the pH values in the equilibrium solutions for Cr(III) ions are shown in Fig. 3.

It was observed that the uptake of Cr(III) ions by ferrihydrite increased with an increase in initial Cr concentration and depended on both the pH of the solution and type of anion except for the solutions at C_0_ < 50 mg L^−1^ and pH 5.0 (Fig. 3A). At 0.1 < C_0_ < 50 mg L^−1^ and pH 5.0 the adsorption capacity of ferrihydrite did not depend on the type of anion and ranged from 0.078 ± 0.003 mg g^−1^ to 20.9 ± 1.4 mg g^−1^ (*RE* = 94.2 ± 1.39%–42.3 ± 1.41%). At higher initial Cr concentrations, a higher affinity for the surface of ferrihydrite for sulfate solutions than for chloride solutions occurred, up to 59.2 ± 1.71 mg g^−1^ and 49 ± 2.5 mg g^−1^, respectively. With a decrease in the pH of the initial solution to 3.0, the adsorption capacity of ferrihydrite for Cr(III) decreased over the whole range of initial concentrations. The maximum adsorption capacity of ferrihydrite decreased by 39.9% to 35.6 ± 2.27 mg g^−1^ from sulfate solutions and by 42.4% to 28.2 ± 1.59 mg g^−1^ from chloride solutions. The adsorption capacities of ferrihydrite for Cr(III) ions decreased in the following order: *q* (SO${}_{4}^{2-}$, pH 5.0) > *q* (Cl^−^, pH 5.0) > *q* (SO${}_{4}^{2-}$, pH 3.0) > *q* (Cl^−^, pH 3.0). The results showed that with increasing initial concentration of Cr(III), the removal efficiency decreased from 98.8 ± 0.52% to 2.69 ± 0.15%. The decrease in the removal efficiency can be explained by the fact that the adsorbents have a limited number of active sites that would have become saturated above a certain concentration (Bedemo, Chandravanshi & Zewge, 2016).

The adsorption of Cr(III) ions from sulfate solutions at pH 5.0 and pH 3.0 occurred at pH 5.79–3.69 and 4.02–2.94, respectively (Fig. 3B). The pH values in the equilibrium solutions of chlorides changed in the ranges of 5.00–4.02 and 3.55–3.07, respectively (Fig. 3B). The pH of the equilibrium solution depends on the buffering capacity of the adsorbent, the pH of the equilibrium solution and the chemical reactions accompanying the metal ion binding process. Ferrihydrite was characterized by low cation exchange capacity, lack of basic exchangeable cations, low buffering capacity and poor ability to oppose pH changes, especially at their lower values. With an increase in the initial concentration of Cr(III) from 0.1 to 10 mg L^−1^, the pH of the equilibrium solution was constant, higher than that of the initial solution (maximum 5.8 for SO${}_{4}^{2-}$, pH 5.0), and then decreased to maximum 2.71 (Cl^−^, pH 3.0). The results indicated that the adsorption process may be accompanied by precipitation of chromium hydroxide Cr(OH)_3_ for sulfate solutions at initial pH 5.0 and initial Cr concentration of 0.1–10 mg L^−1^ (Pieczara & Rzepa, 2016; Paja̧k et al., 2018). The presence of H^+^ ions had significant influence on the adsorption capacity of the adsorbent at initial concentrations above 50 mg L^−1^ (Fig. 3B) by affecting surface properties of mineral and protonation/deprotonation reactions of amphoteric surface hydroxyl groups as well as speciation of Cr(III).

**Table 1:** The characteristics of the texture and physico-chemical properties of studied ferrihydrite.

Properties	Specific surface area (m^2^ g^−1^)	Total pore volume (cm^3^ g^−1^)	Pore diameter (nm)	pH_PZC_	pH	CEC (mmol_+_ g^−1^)
Values	296	0.175	1.89	7.35	3.90	0.093

### Adsorption of Cr(VI) ions onto ferrihydrite

**Figure 4:**
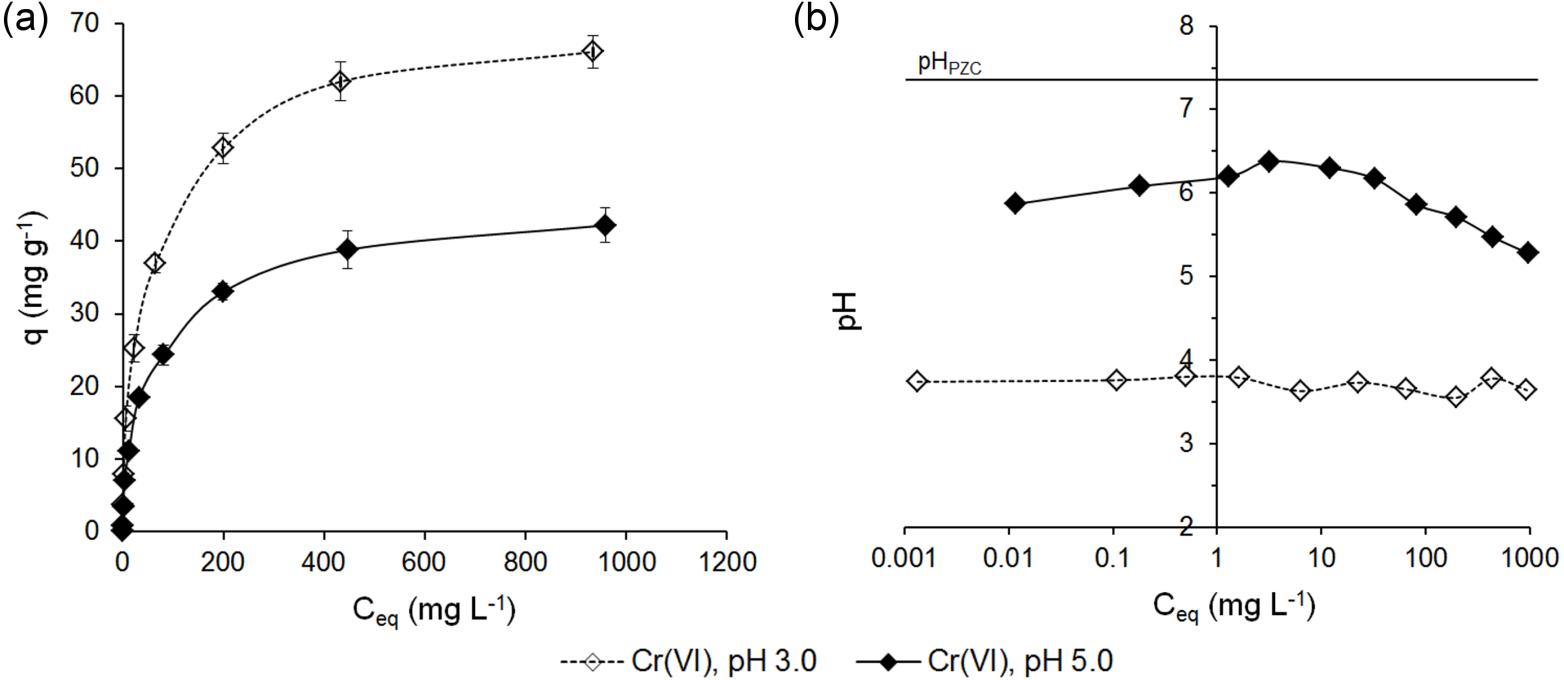
Adsorption of Cr(VI) ions on ferrihydrite (A) and the pH values in the equilibrium solutions (B).

The adsorption of Cr(VI) ions on ferrihydrite (*q* = f(*C*_*eq*_)) and the pHs of the equilibrium solutions are shown in Fig. 4.

The results showed that in contrast to the adsorption of Cr(III) ions, a higher affinity of Cr(VI) ions for the surface of ferrihydrite from the solutions at pH 3.0 than pH 5.0 was observed.

The uptake of Cr(VI) ions from the solutions at pH 3.0 and initial concentrations in the range of 0.1–1,000 mg L^−1^ increased from 0.118 ± 0.004 mg g^−1^ to 66.1 ± 2.36 mg g^−1^ (*RE* = 98.7 ± 0.58% –6.61 ± 0.24%). However, from the solution at pH 5.0 the adsorption of Cr(VI) was lower by 16.9–36.2% depending on the initial concentration and it ranged from 0.098 ± 0.001 mg g^−1^ to 42.2 ± 1.3 mg g^−1^ (*RE* = 91.0 ± 2.7% –4.22 ± 0.13%).

The adsorption of Cr(VI) ions from the solutions at pH 3.0 occurred at practically constant pH values in the range of 3.74–3.64. The pH values in the equilibrium solutions for the adsorption from the solutions at pH 5.0 decreased at higher initial concentration and changed at 6.61–5.28 (Fig. 4B). These observations are in accordance with Cr(VI) studies of *Pieczara & Rzepa (2016)* onto ferrihydrite. Authors suggested that the final pH decrease might cause higher adsorption capacities in more concentrated solutions.

### Isotherm data analysis

The parameters estimated from the Freundlich, Langmuir, Dubinin-Radushkevich (D-R) models using nonlinear and linear regression analysis and Sips model using nonlinear regression, as well as the determination coefficient (*R*^2^) and the error functions (*SSE*, *RMSE* and *χ*^2^) are presented in Table 2.

**Table 2:** The isotherm parameters and error functions for the adsorption of Cr(III) and Cr(VI) ions.

	Cr(III)-SO_4_				Cr(III)-Cl				Cr(VI)			
	pH = 3		pH = 5		pH = 3		pH = 5		pH = 3		pH = 5	
	linear	nonlinear	linear	nonlinear	linear	nonlinear	linear	nonlinear	linear	nonlinear	linear	nonlinear
*Freundlich Isotherm*												
1/*n*_*F*_	0.4282	0.3795	0.5180	0.3334	0.5140	0.5370	0.5793	0.3181	0.5028	0.2892	0.5200	0.2959
*K*_*F*_ (mg g^−1^(L mg^−1^) ^1∕^^*nF*^)	2.086	2.650	2.875	6.663	0.6820	0.7144	1.883	6.168	3.836	10.08	2.172	6.061
*R*^2^	0.9843	0.9931	0.9719	0.9855	0.9464	0.9842	0.9567	0.9684	0.9641	0.9719	0.9561	0.9704
*SSE*	–	9.758	–	62.95	–	13.66	–	97.94	–	167.6	–	67.55
*RMSE*	–	0.9878	–	2.509	–	1.169	–	3.130	–	4.094	–	2.599
*χ*^2^	–	1.618	–	6.441	–	2.421	–	8.961	–	11.04	–	6.995
*Langmuir Isotherm*												
*q*_*exp*_ (mg g^−1^)	35.90	35.90	58.60	58.60	28.20	28.20	49.00	49.00	66.10	66.10	42.20	42.20
*q*_*max*_ (mg g^−1^)	36.23	38.18	59.52	59.74	32.15	37.77	50.25	49.75	66.67	66.23	42.73	42.24
*K*_*L*_ (L mg^−1^×10^−3^)	15.13	7.790	28.78	16.34	5.074	2.819	28.70	20.32	41.85	25.72	32.76	22.34
*R*_*L*_	0.0592	0.1089	0.0376	0.0643	0.1580	0.2525	0.0350	0.0488	0.0233	0.0374	0.0296	0.0428
*R*^2^	0.9754	0.9765	0.9881	0.9804	0.8850	0.9979	0.9948	0.9841	0.9961	0.9812	0.9953	0.9818
*SSE*	–	33.38	–	85.01	–	1.776	–	49.33	–	112.1	–	41.50
*RMSE*	–	1.827	–	2.916	–	0.4214	–	2.221	–	3.348	–	2.037
*χ*^2^	–	82.12	–	28.88	–	17.33	–	7.687	–	31.10	–	14.50
*Dubinin- Radushkevich Isotherm*												
*β* (mol^2^ kJ^−2^×10^−3^)	3.300	5.558	4.500	4.358	4.400	8.458	5.100	4.216	3.900	3.726	4.600	3.904
*q*_*D*_ (mmol g^−1^×10^−3^)	0.629	1.155	1.836	1.815	0.401	1.231	1.837	1.516	1.892	1.933	1.455	1.244
*E* (kJ mol^−1^)	12.31	9.485	10.54	10.71	10.66	7.689	9.901	10.89	11.32	11.58	10.43	11.32
*R*^2^	0.9614	0.9937	0.9970	0.9994	0.8708	0.9959	0.9951	0.9928	0.9796	0.9949	0.9965	0.9943
*SSE*	–	8.889	–	2.465	–	3.588	–	22.41	–	30.30	–	13.05
*RMSE*	–	0.9428	–	0.4965	–	0.5990	–	1.497	–	1.741	–	1.142
*χ*^2^	–	12.45	–	0.2988	–	22.60	–	1.911	–	1.757	–	1.165
*Sips Isotherm*												
*q*_*max*_ (mg g^−1^)	–	83.59	–	86.06	–	40.67	–	61.51	–	83.73	–	53.14
*K*_*S*_ ((L mg^−1^) ^1∕^^*nS*^)	–	0.0225	–	0.0488	–	0.0036	–	0.0488	–	0.0705	–	0.0607
1/ *n*_*S*_	–	0.5070	–	0.5653	–	0.9285	–	0.6553	–	0.5942	–	0.6135
*R*^2^	–	0.9956	–	0.9997	–	0.9983	–	0.9973	–	0.9985	–	0.9983
*SSE*	–	6.216	–	1.305	–	1.501	–	8.24	–	8.889	–	3.900
*RMSE*	–	0.7884	–	0.3613	–	0.3875	–	0.9076	–	0.9428	–	0.6245
*χ*^2^	–	2.368	–	0.3414	–	10.34	–	0.5233	–	0.6875	–	0.4775

The values of *R*^2^ for all four isotherms, estimated by both linear and nonlinear regression, demonstrated a good quality of fitting of the isotherm equations to the experimental data, except for the values of *R*^2^ obtained for Cr(III)-Cl at pH 3.0 for the Langmuir and Dubinin-Radushkevich isotherms (*R*^2^ < 0.90) (Table 2).

The values of the parameter 1/*n*_*F*_ estimated from the Freundlich isotherm, using both regression methods, for all Fh-Cr systems were lower than 1 and indicated the favorable nature of the adsorption. Similar results were obtained by *Adegoke et al. (2014)* who carried out adsorption studies of Cr(VI) on synthetic hematite nanoparticles. Higher intensity of the adsorption of Cr(III) ions (*K*_*F*_) from sulfate solutions than chloride solutions and at pH 5.0 than pH 3.0 was observed. The adsorption intensity of Cr(VI) ions decreased as the pH of Cr(VI) solutions increased (Table 2). Moreover, the values of the parameter *K*_*F*_ correspond very well with the experimental adsorption capacities of ferrihydrite.

The values of the maximum adsorption capacity (*q*_*max*_) estimated from both forms of the Langmuir model for all Fh-Cr systems were similar or greater than the experimental values and ranged from 37.77 mg g^−1^ to 66.23 mg g^−1^using nonlinear regression and from 32.15 mg g^−1^ to 66.67 mg g^−1^using linear regression. Langmuir constant (*K*_*L*_) related to the adsorption energy of Cr(III) was higher for the adsorption at pH 5.0 than pH 3.0 for both Cr(III)-Cl and Cr(III)-SO_4_ solutions and suggested increasing binding strength of Cr to the ferrihydrite surface as the pH of the solution increased. In contrast, for the adsorption of Cr(VI) ions, the values of *K*_*L*_ decreased with increasing pH, indicating lower affinity of Cr for the ferrihydrite surface at higher pH values. The values of *K*_*L*_ calculated using linear regression analysis were higher than those calculated using nonlinear regression. The values of the parameter *R*_*L*_ indicated that the adsorption process was favorable for all Fh-Cr systems. The decrease in *R*_*L*_ with an increase in the pH indicated that the adsorption was more favorable at higher pH for the Fh-Cr(III) systems and at lower pH for Cr(VI). The values of the parameter *R*_*L*_ less than 1 were also obtained by Kar & Equeenuddin (2019) studying the adsorption behavior of Cr(VI) using natural goethite, and *Bhattacharya et al. (2008)* investigating the adsorption of Cr(VI) onto activated alumina.

The values of the parameter *E* estimated from the Dubinin-Radushkevich model using both regression analysis, were found to be higher than 8 kJ mol^−1^ and indicated that the adsorption was a chemical process, except for the adsorption of Cr(III) ions from chloride solutions at pH 3.0, for which the *E* value estimated using nonlinear regression was 7.689 kJ mol^−1^, what indicated the physical nature of the process.

The parameter 1/*n*_*S*_ estimated from the Sips model deviated from unity and denoted the heterogeneity of the surface of the adsorbent, and its values were higher than the values of the parameter 1/*n*_*F*_ for all Fh-Cr systems. The obtained values of *K*_*S*_ were higher than the values of *K*_*L*_ estimated from the Langmuir isotherm. The values of *K*_*S*_ also showed a positive effect of increasing pH on the adsorption process of Cr(III) ions and decreasing pH on the adsorption of Cr(VI) ions. The maximum adsorption capacities estimated from the Sips isotherm were higher than calculated from the Langmuir equation and ranged between 40.67 mg g^−1^ and 86.06 mg g^−1^.

**Figure 5:**
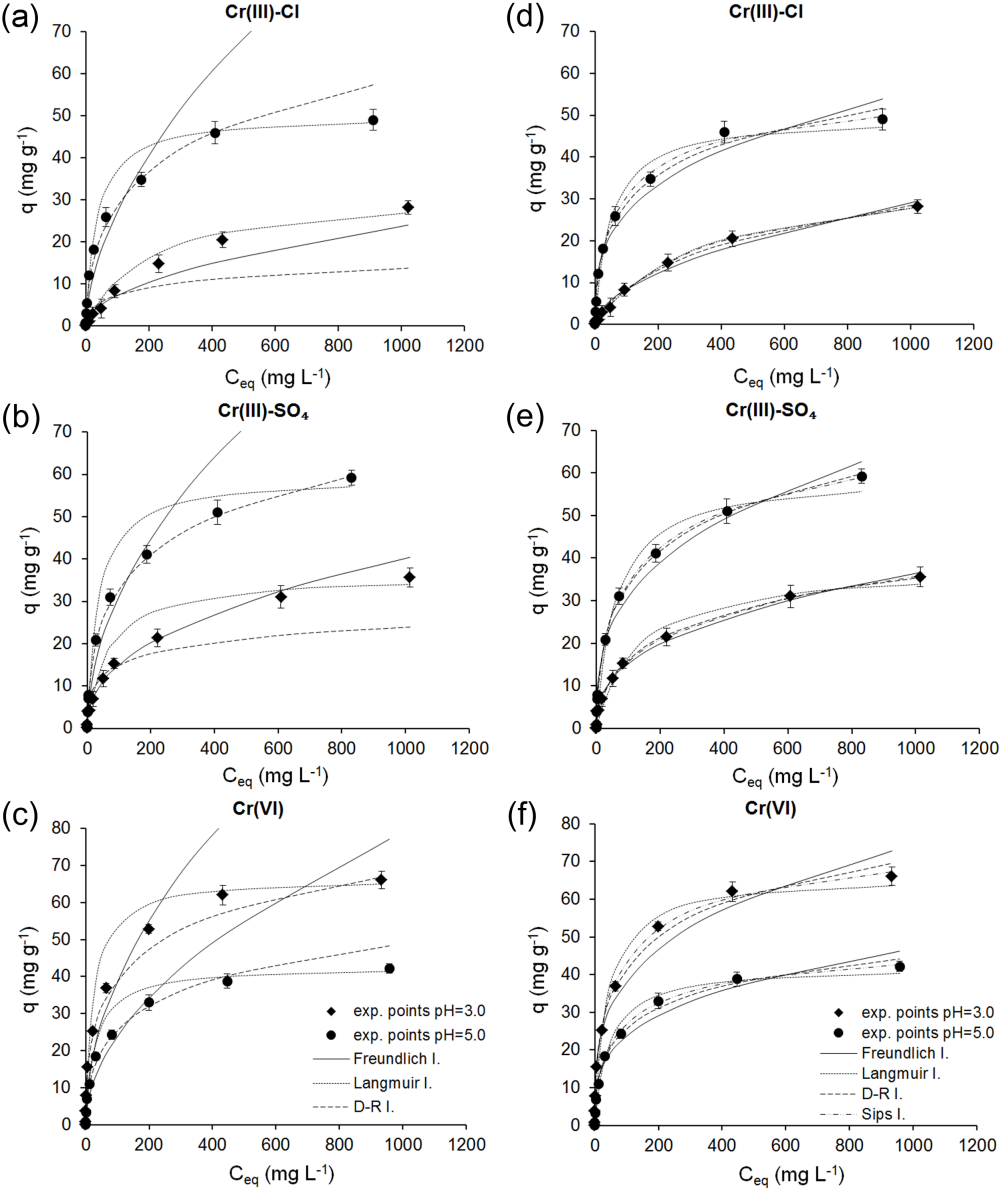
Comparison of experimental and predicted adsorption isotherms obtained using linear (A–C) and nonlinear (D–F) regression analysis for the adsorption of Cr(III) and Cr(VI) ions onto ferrihydrite.

Based on the estimated nonlinear and linear parameters, the theoretical isotherms were plotted, and together with the experimental data are shown in Fig. 5.

Based on the results obtained by linear regression, it was observed that the Freundlich isotherm describes the experimental data in the low initial concentrations, except for the adsorption of Cr(III) ions from sulfate solutions at pH 3.0 when the Freundlich isotherm fits the data best over the whole range of initial concentrations. For the adsorption of Cr(III) ions from chloride and sulfate solutions at pH 5.0 and Cr(VI) ions at both pH the data over the whole range of initial concentrations were best described by the Dubinin-Radushkevich isotherm, and for the adsorption of Cr(III) ions from chloride solutions at pH 3.0 by the Langmuir isotherm.

In Fig. 5 it can be seen that all isotherms estimated using non-linear regression showed a better fit to the experimental data than those estimated using linear regression analysis.

The results obtained by nonlinear regression showed that the Sips isotherm best describes the experimental data over the whole range of initial concentrations of Cr ions. The values of *R*^2^ and analysis of the error functions, *SSE*, *RMSE* and *χ*^2^ (Table 2), also indicated that among four isotherm the three-parameter Sips isotherm is the best-fit model for the adsorption process. The results of the adsorption of Cr(VI) on natural and acid-activated clays obtained by *Khalfa et al. (2016)* also show that three-parameter isotherm model is the best to describe the process.

Since the Sips isotherm is a combination of the Langmuir and Freundlich equations, it can be concluded that the mechanism of Cr binding does not follow an ideal monolayer adsorption, and is therefore more complex. The adsorption capacity obtained from the Sips equation is more realistic than calculated from the Langmuir equation and indicate that different adsorption centers participate in Cr uptake.

The maximum adsorption capacities of various metal oxides including ferrihydrite for the removal of Cr(III) and Cr(VI) are compared in Table 3. However, the direct comparison of adsorption capacities of different adsorbents is difficult due to the various conditions of the adsorption process. Higher values of *q*_*max*_ obtained in this study probably result from much higher initial Cr concentrations.

**Table 3:** Comparison of adsorption capacities of Cr(III) and Cr(VI) of synthesized ferrihydrite with other metal oxides.

Cr species	Adsorbent	Conditions	Maximum adsorption capacity (mg g^−1^)	Ref.
Cr(III)	Ferrihydrite	*C*_0_ 20 µmol L^−1^, adsorbent dose 2.5 g L^−1^, pH 4.5, T 20 °C, t 4 h	5.41	Zhu et al. (2010)
Cr(III)	Nano-magnetite	*C*_0_ 10 mg L^−1^, adsorbent dose 2.5 g L^−1^, pH 4.0, T 21 °C, t 1 h	0.555	Parsons et al. (2014)
Cr(III)	Aluminum oxide hydroxide	*C*_0_ 40 mg L^−1^, adsorbent dose 20 g L^−1^, pH 3.8, T 22 °C, t 1 h	3.36	Bedemo, Chandravanshi & Zewge (2016)
Cr(III)	Birnessite	*C*_0_ 16 mmol L^−1^, adsorbent dose 10 g L^−1^, pH 5.0, T 25 °C, t 24 h	6.63	Qiang, Jiaoyan & Mengzhu (2011)
Cr(III)	Ferrihydrite	*C*_0_ 1,000 mg L^−1^, adsorbent dose 1 g L^−1^, pH 5.0, T 23 °C, t 24 h	59.74	This study
Cr(VI)	Ferrihydrite	*C*_0_ 18.4 mmol L^−1^, adsorbent dose 20 g L^−1^, pH 5.0, T 23 °C, t 24 h	35.13	Pieczara & Rzepa (2016)
Cr(VI)	Goethite	*C*_0_ 25 mg L^−1^, adsorbent dose 10 g L^−1^, pH 2.0, T 23 °C, t 2 h	0.727	Kar & MdEqueenuddin (2019)
Cr(VI)	Goethite	*C*_0_ 16 mg L^−1^, adsorbent dose 4 g L^−1^, pH 8.0, T 23 °C, t 1 h	1.955	Ajouyed et al. (2010)
Cr(VI)	Hematite	*C*_0_ 16 mg L^−1^, adsorbent dose 4 g L^−1^, pH 8.0, T 23 °C, t 3 h	2.299	Ajouyed et al. (2010)
Cr(VI)	*α*-alumina	*C*_0_ 16 mg L^−1^, adsorbent dose 4 g L^−1^, pH 8.0, T 23 °C, t 24 h	2.158	Ajouyed et al. (2010)
Cr(VI)	Nano-magnetite	*C*_0_ 10 mg L^−1^, adsorbent dose 2.5 g L^−1^, pH 4.0, T 21° C, t 1 h	1.705	Parsons et al. (2014)
Cr(VI)	Ferrihydrite	*C*_0_ 1000 mg L^−1^, adsorbent dose 1 g L^−1^, pH 5.0, T 23 °C, t 24 h	66.23	This study

### Bonding strength and mobility of adsorbed Cr(III) and Cr(VI) ions onto ferrihydrite

**Figure 6:**
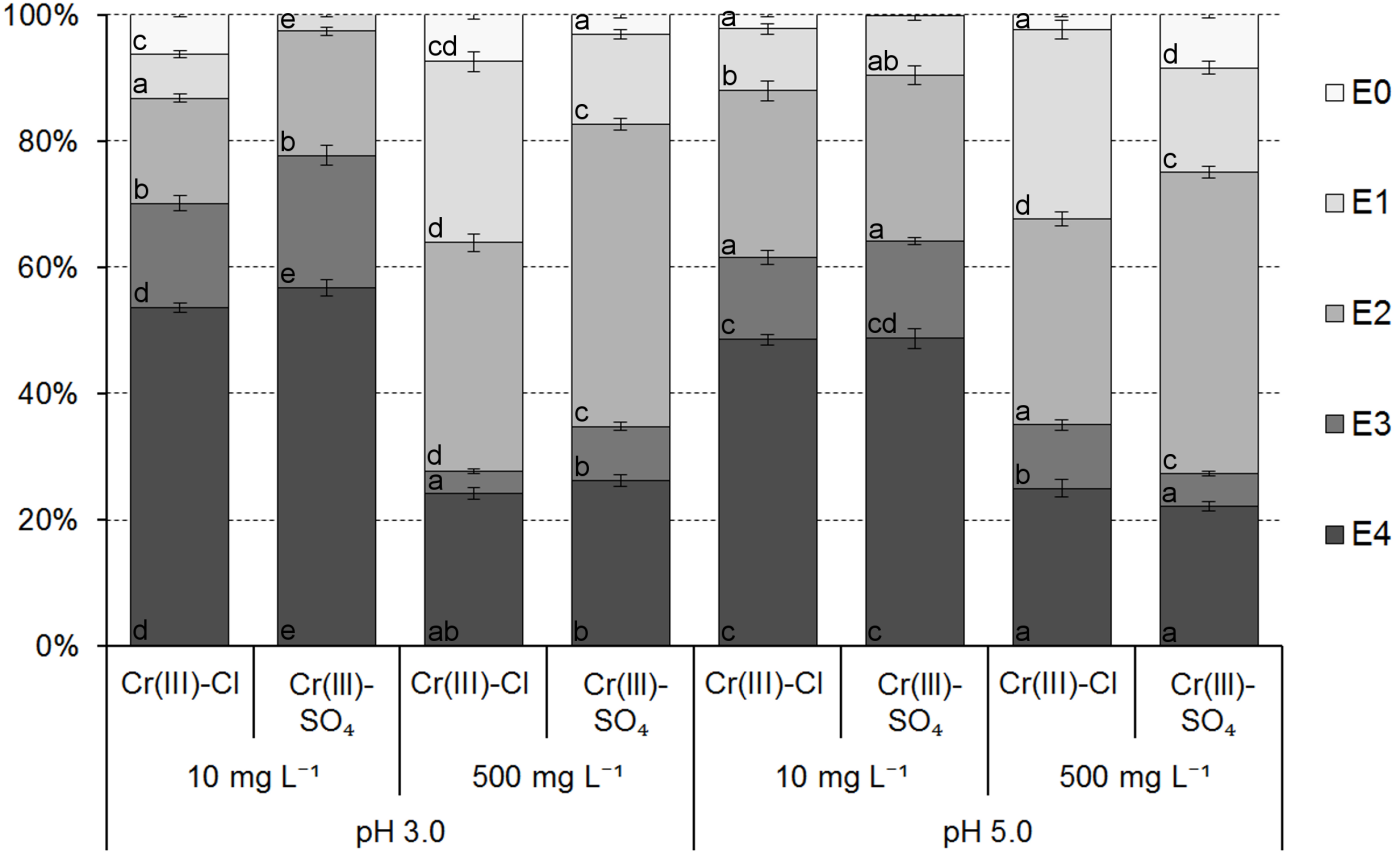
The distribution of Cr(III) ions adsorbed from chloride and sulfate solutions onto ferrihydrite. Data in the individual subsections of each column with the same letter (within stages of extraction) are not significantly different according to Tukey’s HSD test (*P* = 0, 05).

**Figure 7:**
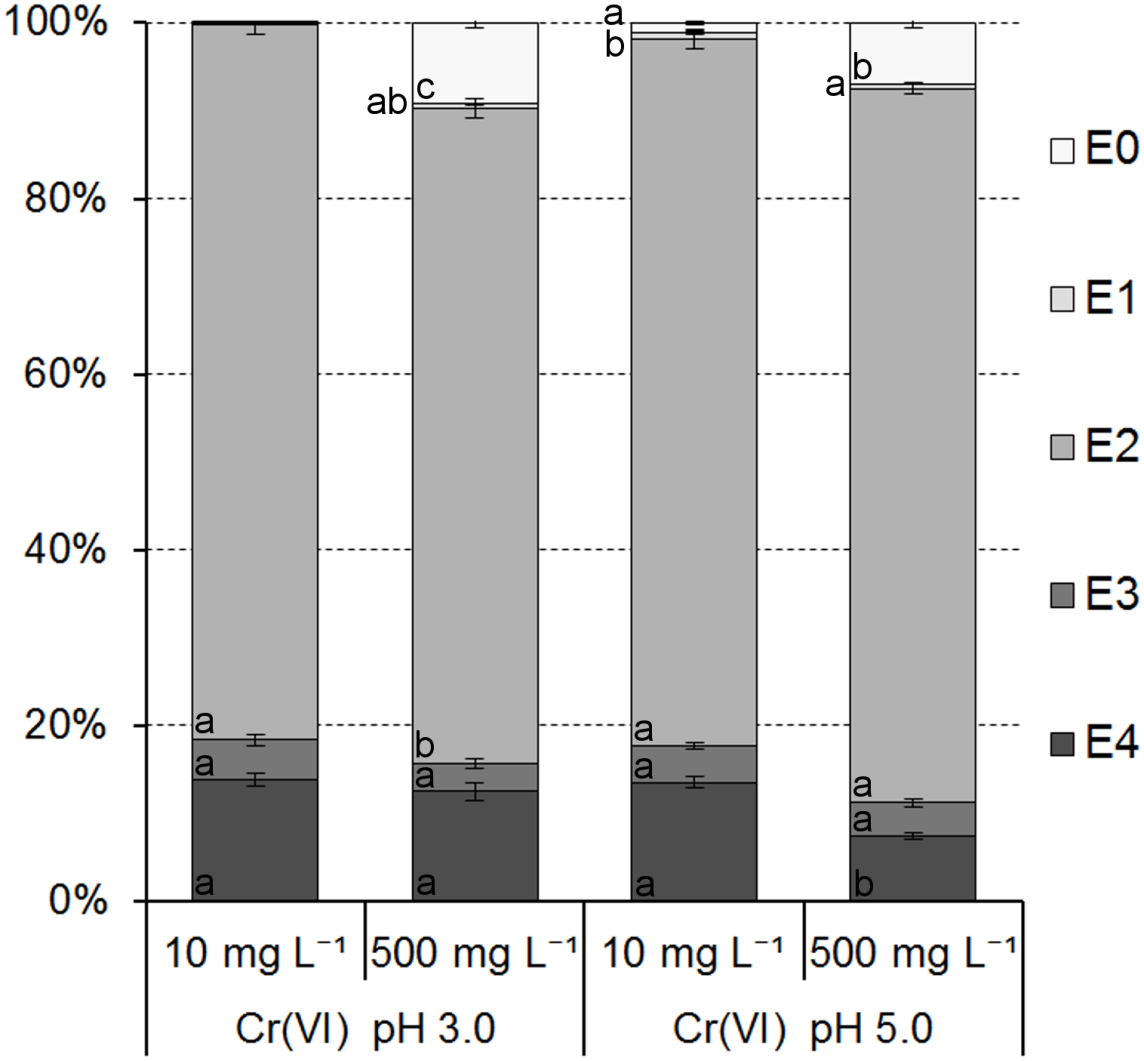
Cr(VI) ions distribution in the ferrihydrite samples. Data in the individual subsections of each column with the same letter (within stages of extraction) are not significantly different according to Tukey’s HSD test (*P* = 0, 05).

The results of the modified BCR sequential extraction of Cr species adsorbed onto the ferrihydrite samples showed that the Cr bonding strength to active sites of ferrihydrite ranged from very weak/weak (E0/E1) to very strong (E4) and are presented in Figs. 6 and 7.

The bonding strength and mobility of Cr(III) ions adsorbed onto the ferrihydrite samples from the chloride and sulfate solutions depended mainly on the initial concentration of Cr in the solution, and less depended on the initial pH of the solution and the type of anion. The effect of pH was greater for lower concentration, while the effect of the type of anion was greater for higher concentration (Fig. 6). An initial concentration of Cr in the solution and the resulting pH in the equilibrium solution had significant effect on the mobility of adsorbed ions due to the fact that the pH values in the equilibrium solutions affected the forms of metal ions occurrence in the solution and the possibility of their binding by individual fractions (Namiesnik & Rabajczyk, 2010).

The results indicated that in the ferrihydrite samples with adsorbed ions from a solution with an initial concentration of 10 mg L^−1^, Cr(III) ions were dominantly associated with the immobile residual fraction (E4) for both initial pH ranging from 48.52 ± 0.87% to 56.76 ± 1.37% of adsorbed ions. The results confirmed the previous conclusion that Cr(III) can precipitate at low concentration. Distribution of Cr in the fraction E3 ranged from 13.11 ± 1.11% (Cl^−^ ions, pH 5.0) to 20.96 ± 1.61% (SO${}_{4}^{2-}$ ions, pH 3.0) and in the fraction E2 –from 16.59 ± 0.65% (Cl^−^ ions, pH 3.0) to 26.34 ± 1.50% (SO${}_{4}^{2-}$ ions, pH 5.0). Additionally, Cr(III) was bound in fractions E0 and E1 from 0.03 ± 0.01% (SO${}_{4}^{2-}$ ions, pH 3.0) to 6.33 ± 0.32% (Cl^−^ ions, pH 3.0) and from 2.59 ± 0.24% (SO${}_{4}^{2-}$ ions, pH 3.0) to 9.82 ± 0.85% (Cl^−^ ions, pH 5.0), respectively. The Cr(III) binding by ferrihydrite from the solutions at *C*_0_ = 500 mg L^−1^ was weaker compared to the solutions at *C*_0_ = 10 mg L^−1^. The residual fraction (E4) was ranged from 22.15 ± 0.78% (SO${}_{4}^{2-}$ ions, pH 5.0) to 26.23 ± 0.94% (SO${}_{4}^{2-}$ ions, pH 3.0) and the fraction E3 –from 3.53 ± 0.39% (Cl^−^ ions, pH 3.0) to 9.08 ± 0.75% (Cl^−^ ions, pH 5.0). Contribution of Cr in other fractions varied in order E2 (29.3 ± 0.95% 47.85 ± 0.95%) > E1 (14.22 ± 0.69%–28.62 ± 1.58%) > E0 (2.14 ± 0.34%–8.41 ± 0.59%). The results indicated that Cr ions adsorbed from chloride solutions were bound by easily mobile (E0 + E1) fractions in larger amounts than from sulfate solutions. The diameter of ions in solutions (0.48 nm (Cr^3+^)–0.90 nm (Cr_3_(OH)${}_{4}^{5+}$) determined from the ChemBio3D Ultra ver. 12.0 was smaller than the average pores diameter and indicated the possibility of Cr ions binding in the ferrihydrite pores.

The distribution of Cr(VI) ions in individual fractions was only slightly affected by an initial concentration of Cr(VI) in the solutions (Fig. 7).

Cr(VI) ions were bound mainly in the fraction E2 (from 74.49 ± 1.10% to 81.55 ± 0.67% of adsorbed ions). Additionally, they were bound in fractions E3 and E4 from 3.24 ± 0.54% to 4.54 ± 0.64% and from 7.47 ± 0.36% to 13.85 ± 0.82%, respectively. Distribution of Cr(VI) in fractions E0 and E1 was very low from 0.11 ± 0.03% to 9.22 ± 0.63% and from 0.03 ± 0.01% to 0.75 ± 0.12%, respectively, and suggested no or very low mobility and bioavailability in contact with an aqueous solutions containing other metals.

## Discussion

### Adsorption of Cr(III) ions on ferrihydrite

A comparison of the pH values in the equilibrium solutions with the point of zero charge of ferrihydrite (pH_*PZC*_ 7.35, Table 1) showed that the adsorption of Cr(III) ions from sulfate and chloride solutions at both pH occurred at pH < pH_*PZC*_over the whole range of initial concentrations (Fig. 3B) and indicated that the surface of ferrihydrite was positively charged according to Reaction Eq. (1): ≡FhOH + H^+^ → FhOH${}_{2}^{+}$. Therefore electrostatic repulsion existed with Cr(III) ions. This phenomenon is being explained by the surface complex formation model indicating the formation of the surface complexes of metal ions with active sites on oxides (Karapınar, 2016).

The adsorption process of Cr(III) ions is pH dependent as the amounts of Cr(III) ions adsorbed onto ferrihydrite increased with an increase of pH. As the pH decreases, the surface becomes more protonated and hence the affinity of cations to ferrihydrite is lower due to electrostatic repulsions between positively charged surface of ferrihydrite and cationic species and competition with protons. With the increasing pH, the surface becomes less protonated and therefore the cations were bound to ferrihydrite in higher amounts. These results are in agreement with the findings of previous studies on the adsorption of Cr(III) and other heavy metal cations onto iron (oxyhydr)oxides (Zhu et al., 2010; *Meng et al., 2012; Parsons et al., 2014*; *Pieczara & Rzepa, 2016)*.

Cr can occur in solution in different forms depending on the pH of the solution and the type of anion. In the presence of chloride anions (non-complexing anions) at a solution pH = 4, Cr forms mainly three ionic species: Cr^3+^, CrOH^2+^ and Cr_2_(OH)${}_{2}^{4+}$. Moreover, at pH > 3.0, with a maximum at pH = 6, trinuclear Cr_3_(OH)${}_{4}^{5+}$ exists. At pH above 5.1 Cr(OH)_3_ precipitates (Fig. S1A).

Cr(III) ions could be possible bound to the surface of ferrihydrite to one or more binding sites in the following Reactions Eqs. (7)–(10):

(7) \begin{eqnarray*}& & \equiv \mathrm{FhOH}+{\mathrm{Cr}}^{3+}={\mathrm{FhOCr}}^{2+}+{\mathrm{H}}^{+}\end{eqnarray*}

(8) \begin{eqnarray*}& & \equiv \mathrm{FhOH}+{\mathrm{Cr(OH)}}^{2+}\rightarrow {\mathrm{FhOCrOH}}^{+}+{\mathrm{H}}^{+}\end{eqnarray*}

(9) \begin{eqnarray*}& & 3\equiv \mathrm{FhOH}+{\mathrm{Cr}}^{3+}=(\mathrm{FhO})_{3}\mathrm{Cr}+3{\mathrm{H}}^{+}\end{eqnarray*}

(10) \begin{eqnarray*}& & 2\equiv \mathrm{FhOH}+\mathrm{Cr}(\mathrm{OH})^{2+}\rightarrow (\mathrm{FhO})_{2}\mathrm{Cr}(\mathrm{OH})+2{\mathrm{H}}^{+}\end{eqnarray*}

These reactions are accompanied by the release of the H^+^ ions, which causes the solution to acidify, which has been confirmed experimentally (Fig. 3B).

However, in the presence of sulfate(VI) anions (complexing anions) in solution, apart from Cr^3+^, CrSO${}_{4}^{+}$ is formed (Fig. S1B). Sulfates(VI) have impact on different Cr speciation forms over wide range of pH. The presence of SO${}_{4}^{2-}$ ions in the solution suggests that Cr could be bound by hydrogen bonds, in addition to Reactions Eqs. (7) to (10), as follows:

(11) \begin{eqnarray*}\equiv \mathrm{FhOH}+{\mathrm{CrSO}}_{4}^{+}\rightarrow \equiv \mathrm{FhO}-\mathrm{H}-----\mathrm{O}-{\mathrm{SO}}_{3}\mathrm{Cr}.\end{eqnarray*}

The formation of hydrogen bonds between –OH groups from ferrihydrite and –O from SO${}_{4}^{2-}$ groups may cause higher adsorption of Cr(III) ions from sulfate than chloride solutions. Moreover, the K^+^ ions originating from the K_2_CrO_4_ salt or from KOH solution used to adjust the initial pH of the solutions does not compete with Cr(III) for the adsorption because the ion hydration energy (−4,370 kJ mol^−1^ for Cr(III) and −320 kJ mol^−1^ for K^+^ ions) indicated that the K^+^ ion will be more hydrolyzed than Cr(III) and affected the Cr(III) adsorption very weak (Smith, 1977; Igwe & Abia, 2007). At the same time Cr ionic valence is higher (Fig. S1) than that of K and show higher affinity for adsorption.

### Adsorption of Cr(VI) ions onto ferrihydrite

As can be seen in Fig. 4B, the adsorption of Cr(VI) ions proceeded at pH < pH_*PZC*_, therefore the surface of ferrihydrite had a positive charge and could easily bound Cr(VI) anions.

The process was strongly pH dependent what suggest that the adsorption of Cr(VI) is mainly dominated by surface complexation (Ajouyed et al., 2010). Cr(VI) is present in aqueous solutions in forms of HCrO${}_{4}^{2-}$ and dichromate Cr_2_O${}_{7}^{2-}$, and as H_2_CrO_4_ at pH below 1 and CrO${}_{4}^{2-}$ at pH above 4.5 (Fig. S2). Previous studies have shown that chromate binding onto ferrihydrite and other iron (oxyhydr)oxides occurs with the formation of a monodentate and bidentate inner-sphere complexes (Fendorf et al., 1997; Veselská et al., 2016) and outer-sphere complexes (Zachara et al., 1987). Their proportion in ions binding is dependent on the parameters such as the pH, surface coverage, ionic strength and presence of Al in the structure. In general, the formation of bidentate complexes is favored by lower pH and higher surface coverage, while monodentate formation is favored when the surface coverage is low, either due to the presence of insufficient positive charge on the surface (high pH) or low adsorbent concentration. Outer-sphere complexes form in the presence of Al impurities within the crystal and it was found that in pure Fe-ferrihydrite they constitute less than 5% (Kubicki et al., 2018). Therefore, it can be concluded that chromate forms a combination of monodentate and bidentate inner-sphere complexes. According to Al Mamun et al. (2017), hydrogen chromate (HCrO${}_{4}^{-}$) is more strongly adsorbed and it is the dominant surface species for iron or aluminum oxide.

The different forms of Cr(VI) ions in aqueous solutions are given by the following equilibrium Eq. (12) (Ajouyed et al., 2010) and Eq. (13) (Al Mamun et al., 2017):

(12) \begin{eqnarray*}{\mathrm{Cr}}_{2}{\mathrm{O}}_{7}^{2-}+{\mathrm{H}}_{2}\mathrm{O}\Leftrightarrow {\mathrm{2HCrO}}_{4}^{-} \log \nolimits K=-2.2\end{eqnarray*}

(13) \begin{eqnarray*}{\mathrm{HCrO}}_{4}^{-}\Leftrightarrow {\mathrm{CrO}}_{4}^{2-}+{\mathrm{H}}^{+} \log \nolimits K=-6.51\end{eqnarray*}

Possible reactions of Cr(VI) ions binding onto the surface of ferrihydrite are shown in Reactions Eqs. (14) and (15) for the formation of monodentate and bidentate complexes, respectively:

(14) \begin{eqnarray*}\equiv {\mathrm{FhOH}}_{2}^{+}+{\mathrm{HCrO}}_{4}^{-}\rightarrow \equiv {\mathrm{FhHCrO}}_{4}+{\mathrm{H}}_{2}\mathrm{O}\end{eqnarray*}

(15) \begin{eqnarray*}2\equiv \mathrm{FhOH}+{\mathrm{HCrO}}_{4}^{-}+{\mathrm{H}}^{+}\rightarrow (\equiv \mathrm{FhO})_{2}{\mathrm{CrO}}_{2}+2{\mathrm{H}}_{2}\mathrm{O}.\end{eqnarray*}

The pH of the solution is an important factor affecting the adsorption capacity due to its impact on the surface properties (surface charge) of the adsorbent, as well as on the ionic form of Cr in the solution. The adsorption of Cr(VI) ions increased with decreasing pH of the solution. A similar behavior has been reported in other studies on chromate adsorption onto ferrihydrite (Tzou, Wang & Loeppert, 2003; Al Mamun et al., 2017). The increase of H^+^ ions at low pH values, neutralize the negatively charged surface of the adsorbents. Higher adsorption is the result of strong electrostatic attraction between the positively charged surface of the adsorbents and Cr(VI) anions. With the increasing pH the number of negatively charged sites increases. The adsorption is lower due to the electrostatic repulsion between the negatively charged surface and Cr(VI) ions and the competition from OH^−^ ions for the adsorption sites (Ajouyed et al., 2010).

Moreover, the Eh values (647.3 and 538.8 mV at pH 3.0 and 5.0, respectively, *C*_0_ = 1,000 mg L^−1^) in the equilibrium solutions were lower than the Eh values in the initial solutions (727.5 mV and 609 mV, respectively) but indicated that there were no reduction conditions and Cr was bound as Cr(VI).

At the same time, radius of Cr(III) (0.0615 nm) and of Cr(VI) (0.044 nm) indicates that both ion forms can enter the Fh-micropores (diameter of 1.89 nm), thereby increasing the adsorption efficiency, especially at high concentrations of Cr ions.

## Conclusions

A batch method was used to study the adsorption of Cr(III) and Cr(VI) ions from aqueous solutions onto synthesized ferrihydrite and the effect of an initial Cr concentration, pH, and the type of anion on the adsorption capacity of ferrihydrite was investigated.

Ferrihydrite was found to be very effective adsorbent for removal all studied Cr species from aqueous solutions. It was found that the adsorption process depended on the oxidation state of Cr. The affinity of Cr(III) ions for the ferrihydrite surface depended on both the type of anion and pH of the solution and the maximum adsorption capacities decreased as follows: *q* (SO${}_{4}^{2-}$, pH 5.0) > *q* (SO${}_{4}^{2-}$, pH 3.0) > *q* (Cl^−^, pH 5.0) > *q* (Cl^−^, pH 3.0), and were found to be 86.06 mg g^−1^, 83.59 mg g^−1^, 61.51 mg g^−1^ and 40.67 mg g^−1^, respectively. For the adsorption of Cr(III) ions, a higher affinity for the surface of ferrihydrite occurred from sulfate solutions than from chloride solutions and from the solutions at pH 5.0 than pH 3.0. The presence of SO${}_{4}^{2-}$ ions suggests the Cr(III) ions were bound additionally by hydrogen bonds. The amount of adsorbed Cr(VI) decreased as the pH increased and was 83.73 mg g^−1^ at pH 3.0 and 53.14 mg g^−1^ at pH 5.0. The adsorption process of Cr species was pH dependent, and the ions were bound to the surface of ferrihydrite by the formation of the surface complexes of Cr ions with active sites on ferrihydrite.

It was found that the Sips isotherm model, composed of the Langmuir and Freundlich equations, described the adsorption process best and these results suggested that the mechanism of Cr binding does not follow the ideal monolayer adsorption and is more complex.

Studies on the mobility of the adsorbed Cr ions onto ferrihydrite showed that Cr(III) can precipitate at low initial concentration. On the other hand, at an initial concentration of 500 mg L^−1^ Cr(III) ions were bound in mobile forms (E0 + E1) in the range of 17.34–36%. On the contrary, Cr(VI) ions were bound mainly in the reducible fraction (E2) and distribution of Cr(VI) in mobile fractions (E0 + E1) was very low, from 0.14% to 9.97%, and suggested no or very low mobility and bioavailability in contact with an aqueous solutions containing other metals.

The presence of Fe (oxyhydr)oxides in soil and sediments increases their adsorption capacity for Cr, in particular for hexavalent Cr in an acid environment due to their properties (high pH_*PZC*_).

##  Supplemental Information

10.7717/peerj.9324/supp-1 Supplemental Information 1 Supplemental Figures and Tables

10.7717/peerj.9324/supp-2 Supplemental Information 2 Raw data: the results of the adsorption and BCR sequential extraction experiments

## Additional Information and Declarations

### Data Availability

The following information was supplied regarding data availability: The raw measurements and the raw data are available in the Supplementary Files.
