# Supplementary material for: Adsorption and bonding strength of chromium species by ferrihydrite from acidic aqueous solutions
Source: PeerJ. 2020 Jun 11;8:e9324. doi: 10.7717/peerj.9324 (PMC7293855; doi:10.7717/peerj.9324)
Supplement: Supplemental Information 1 [file peerj-08-9324-s001.docx]

Supplementary Information for

Adsorption and bonding strength of chromium species by ferrihydrite from acidic aqueous solutions

Agnieszka Dzieniszewska, Joanna Kyziol-Komosinska, Magdalena Pająk

Institute of Environmental Engineering Polish Academy of Sciences,
34 M. Skłodowska-Curie St., 41-819 Zabrze, Poland;

Submitted to PeerJ

**This supporting information has 5 pages, including 3 tables and 2 figures**

**Table S1:**

**List of adsorption isotherm models.**

| Adsorption isotherms models | | | | |
| --- | --- | --- | --- | --- |
| Isotherm | Isotherm form | Equation number | Parameters | Ref. |
| Freundlich | Nonlinear  $q=K_{F}\cdot{C_{eq}}^{1/{n_{F}}}$  Linear  $logq=logK_{F}\cdot\frac{1}{n}logC_{eq}$ | (S1.1)  (S1.2) | *K_F_* – Freundlich equilibrium constant related to the adsorption capacity and adsorption intensity of the system (mg g^-1^(L mg^-1^)^1/^*^nF^*),  *1/n_F_* – expresses favorability of adsorption (dimensionless) | *Freundlich, 1906* |
| Langmuir | Nonlinear  $q=\frac{q_{max}K_{L}C_{eq}}{1+K_{L}C_{eq}}$  Linear  $\frac{C_{eq}}{q}=\frac{C_{eq}}{q_{max}}+\frac{1}{K_{L}q_{max}}$ | (S2.1)  (S2.2) | *q_max_* – maximum adsorption capacity  (mg g^-1^),  *K_L_* – Langmuir constant related to the affinity of the binding sites and the energy of adsorption (L mg^-1^) | *Langmuir, 1916* |
| Dubinin-Radushkevich | Nonlinear  $q=q_{D}\cdot exp\left( -\beta\varepsilon^{2} \right)$  Linear  $lnq=lnq_{D}-\beta\varepsilon^{2}$ | (S3.1)  (S3.2) | *q_D_* – theoretical saturation capacity  (mmol g^-1^),  *β* – constant related to the adsorption energy (mol^2^ kJ^-2^),  *ε* – Polanyi potential, $\varepsilon=RTln\left( 1+1/{C_{eq}} \right)$ (S4), *R* – gas constant (J mol^-1^ K^-1^), *T* – temperature (K) | *Dubinin, 1960* |
| Sips | Nonlinear  $q=\frac{q_{max}K_{S}{C_{eq}}^{1/{n_{S}}}}{1+K_{S}{C_{eq}}^{1/{n_{S}}}}$ | (S5) | *K_S_* – Sips constant related with affinity constant ((L mg^-1^)^1/^*^nS^*),  1/*n_S_* – Sips exponent which represents the surface heterogeneity (dimensionless) | *Sips, 1948* |

**Table S2:**

**List of error functions.**

| Error functions | | | | |
| --- | --- | --- | --- | --- |
| Abbreviation | Definition/expression | Equation number | Parameters | Ref. |
| SSE | $\sum_{i=1}^{n} \left( q_{e,cal}-q_{e,exp} \right)_{i}^{2}$ | (S6) | *q_e,cal_* – calculated value of the adsorption capacity,  *q_e,exp_* – experimental value of the adsorption capacity,  *n* – number of observations in the experimental data | *Foo & Hameed, 2010* |
| *RMSE* | $\sqrt{\frac{1}{n}\sum_{i=1}^{n} \left( q_{e,exp}-q_{e,cal} \right)_{i}^{2}}$ | (S7) |  | *Terdputtakun et al., 2017* |
| *χ^2^* | $\sum_{i=1}^{n} \frac{\left( q_{e,exp}-q_{e,cal} \right)^{2}}{q_{e,cal}}$ | (S8) |  | *Terdputtakun et al., 2017* |

**Table S3:**

**Modified BCR sequential extraction scheme.**

| Stage of extraction | Fraction | Extractant | Conditions |
| --- | --- | --- | --- |
| E0 | Soluble in water | Double distilled H_2_O | S:L = 1:40, 2 h, T = 20 °C |
| E1 | (Acid soluble/  Exchangeable) – bound to carbonates | 0.11 M CH_3_COOH (pH 2.85) | S:L = 1:40, 16 h, T = 20 °C |
| E2 | Reducible – bound to Mn  and Fe oxides | 0.5 M NH_2_OH·HCl (pH 1.5) | S:L = 1:40, 16 h, T = 20 °C |
| E3 | Oxidizable – bound to  organic matter and sulfides | 8.8 M H_2_O_2_,  then 1 M CH_3_COONH_4_ (pH 2.0) | S:L = 1:10, 1 h, T = 80 °C  S:L = 1:50, 16 h, T = 20 °C |
| E4 | Residual | Aqua regia (HCl/HNO_3_ 3/1),  then 0.1 M HNO_3_ | S:L = 1:25, T = 80 °C  S:L = 1:50 |

Diagram of chromium speciation for Cr(III) from chloride and sulfate solution as well as for Cr(VI) at *C*_0_ = 1000 mg L^-1^ was generated using the software Visual MINTEQ version 3.1 [http://vminteq.lwr.kth.se/download/].


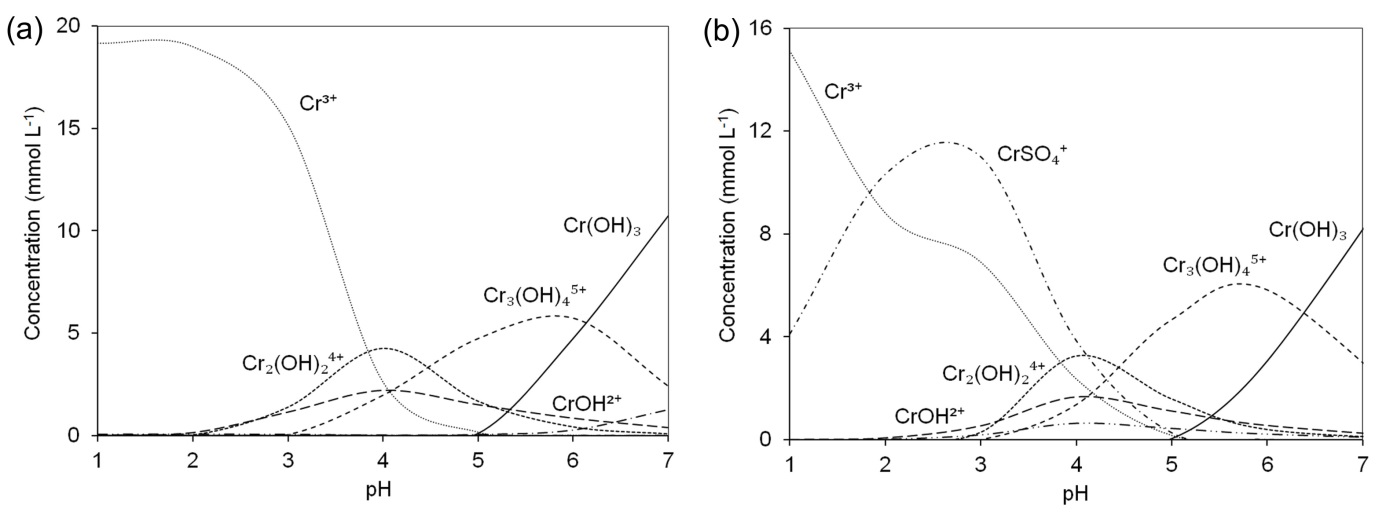


**Figure S1: Cr(III) speciation in chloride (A) and sulfate (B) solutions at *C*_0_ = 1000 mgCr L^-1^ as a function of pH.**


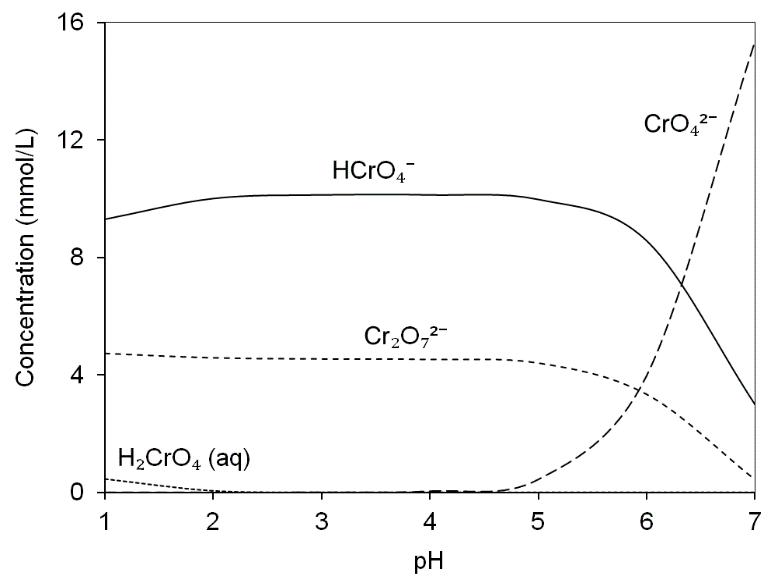


**Figure S2: Cr(VI) speciation depending on the pH in the solution at *C*_0_ = 1000 mgCr L^-1^.**
